# Meeting Report: Experimental and Evolutionary Approaches to Yeast and Other Organisms 2018

**DOI:** 10.1534/g3.118.201003

**Published:** 2019-02-12

**Authors:** David Gresham, Giulia Rancati

**Affiliations:** * Center for Genomics and Systems Biology, Department of Biology, New York University, 10012 and; † Institute for Medical Biology, A*STAR, Singapore 138648

## Abstract

The 2018 European Molecular Biology Laboratory (EMBL) Experimental and Evolutionary Approaches to Yeast and Other Organisms conference brought together researchers addressing fundamental questions in microbial evolutionary systems biology. Topics spanning evolution in the wild and the lab to molecular mechanisms of adaptation and ecological interactions between species were covered.

Every two years, biologists who span disciplines from evolutionary to synthetic biology and work with organisms ranging from yeast to humans, congregate above the German town of Heidelberg at the European Molecular Biology Laboratory (EMBL) to report on recent progress. In October 2018, the 5^th^ biennial meeting organized by Maitreya Dunham, Judith Berman, Jun-Yi Leu and Kiran Patil, continued the tradition with an outstanding lineup of speakers and poster presenters over a four-day period. Here, we highlight key themes that emerged from this meeting and summarize new and unpublished results that were presented. For the sake of brevity, we describe only oral presentations even though the quality of the poster presentations was excellent. We identified five major themes that encompassed the majority of presentations.

## The Evolutionary History of Yeast

The recent publication of genome sequences and phenotypes of 1,011 *S. cerevisiae* strains has provided a great resource for understanding the evolutionary history of this species (Peter *et al.* 2018). Dissection of genetic diversity in the analyzed strains has revealed that several segregating genes in the global yeast population are not present in the S288c lab reference strain. Specifically, as described by Anne Friedrich, while 2,908 genes are variably present across different *S. cerevisiae* strains, 1,712 genes are absent in the reference genome. This genetic variability in gene content is also present when looking at the “pangenome” of 6 other Saccharomycotina species which also have both a core and accessory genome. While this poses challenges to genotype-phenotype mapping, it suggests that yeast has an open pangenome possibly due to extensive interspecific hybridization between closely related species. The idea that genetic variation could result from hybridization events was also put forward by Anne Clark who described the history of introgressed sequences from *S. paradoxus* in 93 *S. cerevisiae* strains isolated from different ecological niches. The hypothesized 3,000 introgressed regions are distributed on all chromosomes. While the amount of introgression varies considerably between different strains, it accounts for ∼8% of all polymorphisms. Another path to variation comes from whole genome duplication, an ancient event that shaped the evolutionary history of yeast. Christian Landry described investigation of how gene duplication contributes to genetic robustness by studying the effect of gene duplicates on protein-protein interactions. Surprisingly, rather than providing functional redundancy, duplicates that function as heterodimers become functionally dependent on each other (Diss *et al.* 2017). As we learn more about the past of yeast, the future evolutionary history of yeast looks increasingly unnatural. Jef Boeke provided an update on the continuing work to modify the yeast genome. While efforts to reduce, relocate and modify the yeast genome to achieve inducible genetic flexibility are well on the way as part of the Sc2.0 project, he presented recent data on karyotype engineering to concatenate yeast chromosomes. Remarkably all genetic information in yeast can be faithfully transmitted in one (Shao *et al.* 2018) or two (Luo *et al.* 2018) chromosomes with little apparent fitness cost for the organism. On top of being a remarkable example of eukaryotic genome restructuring, this work suggests that karyotype evolution could have occurred in an ancient single-linear-chromosome organism by several rounds of genome duplications followed by gene loss. As described by Aimée Dudley, the utility of engineering yeast strains also has other applications. Genetic complementation assays in which human disease genes replace their yeast orthologs can be used to study the functional consequences of human genetic variation. Large-scale analysis of allelic variation, so called deep mutational scanning, has emerged as a powerful approach for structure-function analysis and has dramatically expanded the utility of yeast for predicting the effect of human variants of uncertain significance (VUS), by far the largest class of variants detected in clinical sequencing.

Although evolutionary biology benefits from generalizability of principles, sometimes, the exceptions are informative. Ken Wolfe described a fascinating story about the evolutionary origins of the mating type switching endonuclease gene, *HO*, which has no orthologs except in *S. cerevisiae* and is absent from almost all pre-whole genome duplication yeast species. The discovery of WHO genes (Weird HO) in *Torulaspora delbrueckii* points toward a model in which *HO* originated from the only intein-containing gene in yeast, *VMA1*. Its fusion to a zinc finger domain provides the protein sequence specificity and ability to integrate into empty alleles. Another exception was described by Anne-Ruxandra Carvunis, who summarized recent progress in understanding *de novo* gene birth, a process that generates proto-genes without ancestral gene modification. This process was initially thought to be extremely rare but has become a subject of greater interest following the identification of some 2,000 proto-genes by ribosome profiling data (Carvunis *et al.* 2012). Interestingly, 5% of 342 studied protogenes confers beneficial phenotypes upon overexpression. These beneficial proto-genes are enriched in transmembrane domains and have T-rich regions, likely encoding hydrophobic amino acids. In one case, alignment of the proto-gene sequence to other yeasts suggests that the transmembrane domain predated the coding potential.

## Yeast as a model for species hybridization

The tractability of yeast as an experimental system has made it the preeminent tool for studying species hybridization. In an experimental *tour de force*, Jun-Yi Leu reported the construction of chromosomal replacement strains in which each *S. cerevisiae* chromosome has been replaced by the corresponding *S. paradoxus* chromosome. Multiple lines of evidence trace the decreased fitness in these “hybrids” to proteotoxic stress, derived from the inability of proteins encoded from the two different genomes to correctly assemble into complexes. Conversely, hybrids can also display higher fitness than the parental lines, as described by Rike Stelkens, who showed that F1 and F2 strains generated from *S. cerevisiae*/*S. paradoxus* hybrids have increased survival probabilities in stressful conditions. In order to rapidly map species-specific phenotypic differences, Rachel Brem described a recently published (Weiss *et al.* 2018) approach using genome-wide reciprocal hemizygosity testing coupled with random transposon mutagenesis in hybrids. This clever approach is also likely to have utility for identifying causative genes in traditional QTL mapping using F1 hybrids. Large-scale random insertional mutagenesis is clearly the way forward for functional genomics in diverse strains and species. Application of this method to *C. albicans* haploid clinical isolates by Judith Berman led to the definition of the “essentialome” in this difficult to manipulate human pathogen (Segal *et al.* 2018) offering new targets for therapeutic avenues.

## Experimental Approaches to Evolution

Yeast continues to be unparalleled in its utility for tackling evolutionary questions using experimental evolution. Having all the benefits of microbial culturing and a rich toolkit of molecular and genetic methods makes it the ideal organism for experimental evolution. Gavin Sherlock, employing the widely-adopted lineage tracking method previously developed in his lab (Levy *et al.* 2015), reported that evolution in closely related environments causes a fitness trade-off between mutations. The resulting inaccessible space of mutational effects is condition-specific and likely caused by lack of selection. Michael Desai described an extension of this tracking method to extend the ability to monitor evolutionary dynamics using recurrent barcoding. Initial application of this enhanced method provides experimental support for the “traveling wave” model of adaptive mutations in competing lineages (Desai, Fisher, and Murray 2007) and that less fit lineages can “leapfrog” to the front of the wave by acquiring a high fitness mutation. One of us (David Gresham) described the combination of lineage tracking with a reporter system for identifying copy number variants (CNVs) to study the diversity and dynamics of lineages containing CNVs in evolving populations (Lauer *et al.* 2018). This study has revealed that thousands of CNVs compete in large populations, many of which are likely generated by the replication-based mechanism, ODIRA (Brewer *et al.* 2015). CNVs, and other structural genomic changes, are frequently observed in microbial evolution resulting in alteration of gene dosage, but different strains likely respond differently to over-expression of the same genes. To test for natural variation in the effects of gene dosage changes, Audrey Gasch described studies to compare tolerance, and functional effects, of gene overexpression in a panel of wild yeast strains. Results suggest substantial variation in how different lineages tolerate amplification of the same genes, which has implications for the phenotypic landscape to which different evolving strains have access.

The scale of experimental evolution in yeast continues to increase. Jonas Warringer described propagation of 9,000 yeast populations and intriguing evidence for genetic assimilation - during long-term selection mitochondrial function degrades with the degradation remaining as a cellular memory for 8-10 generations after relaxation of the selection. Under prolonged selection, the memory was made permanent by the mitochondrial genome going extinct, a process that was accommodated by chromosome 2, 3 and 5 aneuploidies. One of the holy grails of experimental evolution is to detect the emergence of novel functions, rather than loss or enhancements of existing functions. Following in the wake of results in *E. coli* from the Lenski lab demonstrating evolution of the novel ability to metabolize citrate (Blount *et al.* 2012), efforts are underway to study the acquisition of novel functions in other systems. Maitreya Dunham described experimental evolution studies that result in *SAO1*, a sulfonate transporter, acquiring the novel ability to transport ammonium sulfate. Deriving novel functions from DNA sequence may be much easier than we think as Jeff Gore reminded us of his recently published study about the rapid acquisition of *de novo* promoter activity from random sequence in *E. coli* (Yona, Alm, and Gore 2018). However, the pathway to novel phenotypes may be complex as Balazs Papp told us that selection for suppressors of deleterious mutations can result in alternative high-fitness mutants which rarely display a wild-type morphology. In a case study of adaptive evolution in response to the replacement of the mitotic cohesion subunit with its meiotic counterpart, Yu-Ying Hsieh found recurrent mutations in the CDK8 complex, which resulted in a slower cell cycle progression, improved chromosome pairing and a partially suppressed cohesion defect. Addressing the evolution of novelty using experimental evolution is an area ripe for investigation.

## Molecular mechanisms in an evolutionary context

Evolutionary consequences provide an explanation for why certain phenomena are observed, but understanding the molecular bases of regulatory processes that are critical in the life-history of the organism is required for understanding the details of how evolution works. How organisms respond to stress is of critical importance for understanding how organisms can survive in the wild. Naama Barkai reported new insights into how regulation of expression and noise by *MSN2* and *MSN4* balances the cell’s capacity to respond to stress, while maintaining full growth potential. MSN2 and MSN4 activate the same set of stress responsive targets. Whereas *MSN2* is constitutively expressed at a low level, *MSN4* is expressed noisily and is strongly inducible. This redundant two-factor regulatory system provides a mechanistic solution to the challenge of rapidly responding to transient stress while maintaining the capacity to proliferate in the presence of persistent stress. Non-genetic heterogeneity within a population of genetically identical cells may provide additional mechanisms for survival. Mark Siegal reported the role of *MSN2* and *MSN4* in generating cell-to-cell heterogeneity, which in turn contributes to the ability of the heterogeneous population to survive an acute stress (Li, Giardina, and Siegal 2018).

Studying the molecular mechanisms of regulation requires the generation of new tools. Naomi Ziv described the development of estradiol synthetic inducible systems (McIsaac *et al.* 2013) to recapitulate the regulatory mechanisms underlying the bistable stochastic switching between white and opaque phenotypes in *C. albicans*. Jens Nielsen and Kiran Patil described the use of multi-omics approaches to track the contribution of epigenetic changes to adaptive phenotypes and to highlight the role of metabolite exchange in complex microbial ecosystems, respectively.

Christian Brion described a clever single-cell approach to simultaneously mapping QTL that underlies variation in protein and mRNA abundance of a gene using an extension of the x-QTL based mapping approach (Albert *et al.* 2014). Chris Jakobson summarized his recent QTL study that provides insight into the relationship between genetic variation and quantitative traits using crosses between minimally diverged strains (She and Jarosz 2018). Interestingly, more than half the causative variants at QTL are synonymous or regulatory, suggesting a pervasive role for natural variation in tuning both transcription and translation. However, Ian Ehrenreich provided a sobering reminder of the complexity of genotype-phenotype relationships in his dissection of the multigenic and non-additive genetic basis of colony morphology phenotypes (Lee *et al.* 2018). The generation of heritable variation may also occur in unexpected and nonrandom ways, as Jon Houseley described the formation of circular DNA encoding the *CUP1* gene when cells age in the presence of copper, an interesting extension of his recent report that *CUP1* CNVs are stimulated by the presence of environmental copper (Hull *et al.* 2017).

## Host-parasite interactions

Perhaps the most exciting and novel topic of the meeting was the emerging utility of using fungal systems for studying interactions between species and between hosts and their parasites. Harmit Malik told us about natural variation in the capacity for different yeast strains to maintain the 2 micron plasmid, a selfish genetic element that confers no benefit to the host. QTL mapping between a restrictive and permissive strain identified a single locus on chromosome V that appears to underlie this variation. The elucidation of the causative gene should provide new insight into this poorly understood class of genetic variation. Selfish genetic elements also likely contribute to some of the results that have been observed in experimental evolution populations. Sean Buskirk reported new studies of the role of the killer virus in experimental evolution. While present in the ancestor, killer ability is commonly lost from evolving populations, owing to mutations in the killer virus genome. These populations can subsequently develop sensitivity to killer toxin, leading to a rock-paper-scissor scenario in which evolved populations lose in competition assays with the ancestral genotype. A fascinating talk about the nematode eating fungus, *Arthrobotrys oligospoara*, by Yen-Ping Hsueh highlighted the amazing diversity of fungal lifestyles and their ability to trap nematodes. This fungal species is ubiquitous in the environment and the wild isolates are highly polymorphic in their prey-sensing ability. G-protein signaling plays a major role in sensing ascarosides secreted by nematodes, which induces trap formation by the fungus. Kirsten Nielsen told us that the human pathogen *Cryptoccus neoformans* tries to evade host immune cells and adapt to host lungs by generating titan cells that are up to 100μm in diameter through endoreduplication. These polyploid cells generate daughter cells that are genetically distinct and may be beneficial in the presence of various stresses, including antifungal drugs (Gerstein *et al.*, 2015).

## Conclusion

While all attendees clearly appreciate the value of yeast as a model system for evolutionary biology, the broader gifts to humankind provided by our microbial friend were celebrated through a visit to the local brewery. Our tour guide was informed that he would be accommodating a large group of yeast researchers – surely, the most difficult audience he had encountered in his career – and held up admirably in front of the knowledgeable crowd.

Some of the most exciting work was presented in poster format. We congratulate all poster presenters on their excellent posters. In particular, the five poster winners Michelle Hays, Milo Johnson, Gat Krieger, Marta Lukacisinova and Alex Nguyen Ba exemplified the outstanding quality of poster presentations. We look forward to hearing more from the exciting emerging stories presented in these posters.

The importance of this biennial meeting for the field cannot be overstated. There is no other meeting in the world that matches the depth and breadth of topics relevant to evolution and ecology in fungal species. This is especially true since the American Society for Microbiology’s decision to cancel the highly successful Experimental Microbial Evolution meeting. The organizational aspects of the meeting including the length of the talks, diversity of topics and time allotted for poster sessions are strengths of the meeting. In general, talks and posters reported new and unpublished work, often presented by trainees. The relatively small size of the meeting (130 attendees) truly allows for scientific exchange, discussion and for establishment of new collaborations. Future topics for the 2020 meeting might include microbial ecology, food microbiology and industrial applications. The EMBL meeting has established itself as the preeminent forum for reporting progress in our understanding of microbial evolution and ecology. We look forward to making the pilgrimage to Heidelberg in two years and the continuing evolution of this essential meeting.
